# Prognostic Factors in Adult Patients with Dengue: Developing Risk Scoring Models and Emphasizing Factors Associated with Death ≤7 Days after Illness Onset and ≤3 Days after Presentation

**DOI:** 10.3390/jcm7110396

**Published:** 2018-10-28

**Authors:** Ing-Kit Lee, Chung-Hao Huang, Wen-Chi Huang, Yi-Chun Chen, Ching-Yen Tsai, Ko Chang, Yen-Hsu Chen

**Affiliations:** 1 Division of Infectious Diseases, Department of Internal Medicine, Kaohsiung Chang Gung Memorial Hospital, Kaohsiung 833, Taiwan; heteyland@cgmh.org.tw (W.-C.H.); sonice83@cgmh.org.tw (Y.-C.C.); greenswallow822@yahoo.com.tw (C.-Y.T.); 2 Department of Internal Medicine, Chang Gung University Medical College, Tao-Yuan 330, Taiwan; 3 Division of Infectious Diseases, Department of Internal Medicine, Kaohsiung Medical University Hospital, Kaohsiung 833, Taiwan; locusthao@gmail.com (C.-H.H.); johnsonckk@yahoo.com.tw (K.C.); 4 Department of Internal Medicine, Kaohsiung Municipal Ta-Tung Hospital, Kaohsiung 833, Taiwan; 5 School of Medicine, Graduate Institute of Medicine, Sepsis Research Center, Center of Dengue Fever Control and Research, Kaohsiung Medical University, Kaohsiung 833, Taiwan; 6 Department of Biological Science and Technology, College of Biological Science and Technology, National Chiao Tung University, Hsinchu 302, Taiwan

**Keywords:** severe dengue, mortality, scoring models, leukocytosis, gastrointestinal bleeding

## Abstract

Dengue is a mosquito-borne viral disease that is a threat to global health. However, information relating to mortality ≤7 days after dengue onset and ≤3 days after presentation is limited. We retrospectively analyzed 1086 adults with dengue during a 12-year period. Three scoring models were established: model-1 (death ≤3 days after presentation), model-2 (death ≤7 days after illness onset), and model-3 (overall fatality). In total, 39 patients with fatal dengue were identified, of which 17 and 14 patients died ≤7 days after illness onset and ≤3 days after presentation, respectively. In model-1 (range: 0‒4 points), gastrointestinal bleeding ≤72 h after presentation, thrombocytopenia (<50 × 10^9^ cells/L) at presentation, and acute kidney injury after hospitalization, using a cutoff level of 2 points, exhibited good discrimination (area under the receiver curve (AUC): 0.975) between survivors and non-survivors. In model-2, the significant predictors were gastrointestinal bleeding ≤72 h after presentation, and hemoconcentration and leukocytosis after hospitalization. Model-2 (range: 0–4 points) showed an AUC of 0.974, with a cutoff value of 2 points. The independent factors in model-2 were the predictors of overall mortality (model-3), which include thrombocytopenia (<50 × 10^9^ cells/L) at presentation. Using a cutoff value of 2 points, model-3 (range: 0–7 points) revealed an excellent discrimination between survivors and non-survivors (AUC: 0.963).

## 1. Introduction

Dengue is an important acute mosquito-transmitted disease in humans caused by any of the four serotypes of dengue virus in tropical and subtropical regions worldwide [1]. In the past 50 years, the incidence of dengue has increased by 30-fold, with increasing expansion to previously unaffected countries and areas worldwide [2]. According to the World Health Organization (WHO), dengue has been observed in over 100 countries [3]. Moreover, approximately 50–100 million infections are noted annually, and >3.9 billion individuals are at risk [2]. Travelers, who spread the virus from one country to another, have played a key role in the increased incidence of dengue worldwide [4]. In America, the cumulative number of dengue cases in the last 30 years exceeded 5 million [5].

A wide spectrum of dengue manifestations is observed, ranging from inapparent infection to severe and fatal outcomes [1]. To describe and categorize the diverse clinical manifestations of dengue, the WHO developed a classification system in 1975 and revised said system in 1997 [6]. Nevertheless, the use of the 1997 classification system is not sufficiently inclusive for all of the severe forms of dengue [7]. The 2009 WHO guidelines classify patients into two distinct categories to improve the clinical management of dengue: non-severe dengue (with or without warning signs) and severe dengue [3]. The early diagnosis of dengue allows for timely clinical intervention, which is extremely important in the care of patients with dengue [3,8]. However, dengue is a dynamic and complicated disease resulting from complex immunologic reactions between host genetic factors [9], preexisting comorbidities [10], and viral strains [11], and fatality has been reported in some cases, mainly in elderly individuals, despite a timely diagnosis [12,13]. This is particularly true during the last decade of the dengue epidemic, and the emergence of dengue has been associated with a predominance in adults, rather than in children [14].

The risk factors associated with increased severity and fatal outcomes have been addressed in previous studies [15,16,17,18,19]. A study from Taiwan has shown that old age, respiratory distress, altered consciousness, and thrombocytopenia were significantly associated with the risk of mortality [15]. A study from Brazil reported that age >55 years, gastrointestinal bleeding, hematuria, and thrombocytopenia were independently associated with mortality [16]. Another study presented 1062 dengue-related deaths in both children and adults in Brazil, and Moraes et al. revealed that a high hematocrit level was significantly associated with mortality [17]. A case series from Singapore described shock as the most common cause of death, followed by organ impairment [18]. Of the 11 patients (both children and adults) with fatal dengue in Puerto Rico, 45% and 27% presented with gastrointestinal bleeding and hemoconcentration [19]. In fact, most studies were vigilant in all-cause mortality from dengue. Information regarding the causes and factors related to mortality during the first week after onset of dengue illness and ≤3 days after hospital presentation is limited. Research that investigates reliable clinical parameters for the early prediction of outcome from dengue must be urgently conducted. To overcome these limitations, this retrospective study was conducted. We identified 1086 adult patients with dengue from 2002 to 2015 and identified the risk factors of mortality from dengue. This study aimed to develop risk scoring systems that can be applied clinically to identify those at higher risk for fatality upon arrival and during hospitalization.

## 2. Materials and Methods

### 2.1. Ethics Statement 

The institutional review board of Kaohsiung Chang Gung Memorial Hospital (KSCGMH) and Kaohsiung Medical University Hospital (KMUH) approved the study (document no.: 201801445B0). 

### 2.2. Design, Patients, and Severity of Dengue 

A retrospective study of adult patients (≥18 years) with dengue was conducted at KSCGMH (2500-bed facility) between 2002 and 2015 and at KMUH (1700-bed facility) between 2009 and 2013 in Taiwan. The included patients tested positive based on at least one of the following criteria [3]: (i) positive dengue virus-specific real-time reverse transcription polymerase chain reaction (QuantiTect SYBR Green RT-PCR kit; Qiagen, Hilden, Germany) in the acute-phase serum [20], (ii) a 4-fold increase in dengue virus-specific immunoglobulin (Ig) G antibody in the convalescent serum compared to the acute-phase serum, and/or (iii) detection of dengue virus-specific nonstructural glycoprotein-1 antigen (Bio-Rad Laboratories, Marnes-la-Coquette, France) in the acute-phase serum. Dengue is a notifiable disease in Taiwan. Clinicians notified the Taiwan Center for Disease Control of patients with suspected dengue, and patients’ blood specimens were collected for the central laboratory of the Taiwan Center for Disease Control for the confirmation of dengue. These diagnostic tests were performed and confirmed by the Taiwan Center for Disease Control.

The severity of dengue in this study was based on the criteria of the 2009 WHO dengue classification [3]. The warning signs include abdominal pain or tenderness, persistent vomiting, clinical fluid accumulation (pleural effusion or ascites), mucosal bleed, lethargy or drowsiness, hepatomegaly, and an increase in hematocrit level along with a rapid decrease in platelet count. Severe dengue was defined as severe plasma leakage (hematocrit change > 20%) with shock (systolic blood pressure < 90 mmHg), fluid accumulation with respiratory distress, or severe bleeding or organ impairment.

### 2.3. Definitions

Overall fatality was defined as death due to any cause after hospitalization from dengue. The present study aimed to further stratify fatal cases into two categories: (i) individuals who died ≤7 days after the onset of dengue and (ii) those who died ≤3 days after hospital presentation. Gastrointestinal bleeding was defined as hematemesis and/or passage of tarry or bloody stool [21]. Patients were diagnosed with acute kidney injury if their serum creatinine level increased by 0.3 mg/dL or more within 48 h or elevated to at least 1.5-fold from baseline according to the definition used by Kidney Disease Improving Global Outcomes [22]. Leukocytosis was defined as a peripheral white blood cell count of >10 × 10^9^ cells/L (reference value: 3.0 × 10^9^ to 10 × 10^9^ cells/L). Rhabdomyolysis refers to a 5-fold increase in the serum concentration of creatine phosphokinase (CPK) above the upper limit of the normal range (reference value: 13–130 U/L) with a CPK muscle fraction of >95% and/or an elevated serum myoglobin level [23]. Myocardial injury refers to an increase in serum troponin-I level of >0.5 ng/mL and/or elevated B-type natriuretic peptide level [24]. Hemoconcentration refers to a >20% increase in hematocrit, and it was determined using the following equation: (maximum hematocrit − minimum hematocrit) × 100/minimum hematocrit. Severe hepatitis was defined as plasma aspartate aminotransferase (AST) or alanine aminotransferase (ALT) level of >1000 IU/L [3].

### 2.4. Statistical Analysis

A comparison was made between (i) survivors and non-survivors, (ii) survivors and individuals who died ≤7 days after dengue onset, and (iii) survivors and individuals who died ≤3 days after presentation. A univariate analysis of all demographic, clinical, and laboratory variables available at presentation and during hospitalization was conducted to determine the statistical significance (*P* < 0.05) between the variables and outcomes (survival or death). The Mann–Whitney *U* test and Fisher’s exact test were used to determine the statistical significance of the continuous and categorical variables, respectively. Significant variables with a *P* value < 0.05 in the univariate analyses were used in a multivariate logistic regression model to identify independent demographic, clinical, and laboratory factors associated with death from dengue. We developed a scoring system by assigning the number of points to each independent risk factor by dividing its regression coefficient by the smallest coefficient in the model and rounding this quotient to the nearest whole number [25]. A receiver operating characteristic curve was obtained to evaluate the predictive potential of the score, and the area under the curve (AUC) was identified [26]. The optimal cutoff value was obtained via a receiver operating characteristic (ROC) curve analysis, and its sensitivity and specificity were measured. The Statistical Package for the Social Sciences software version 17.0 (SPSS Inc., Chicago, IL, USA) was used for all data analyses.

## 3. Results

### 3.1. Dengue Epidemic and Patient Characteristics

During the study period (2002–2015), three large dengue outbreaks occurred in 2002, 2014, and 2015 in Taiwan [27,28]. Dengue is not considered endemic in Taiwan; therefore, physicians are not experienced in managing such cases, and, before 2014, the treatment of dengue depended mainly on the clinical practice for the treatment of febrile illness. During the dengue outbreak in 2014 and 2015, the application of the 2009 WHO guideline for dengue diagnosis and treatment was implemented. Recommendations from the 2009 guideline regarding fluid replacement therapy were modified according to different clinical settings because most patients during the epidemic in 2014 and 2015 were elderly individuals.

In total, 1068 patients comprising 513 men and 555 women, with a median age of 52 (range: 18–91) years, with laboratory-confirmed dengue viral infection were assessed. The overall case fatality rate was 3.6% (39 of 1068 patients) (Table 1).

### 3.2. Clinical Features of Fatal Patients

**Figure 1:**
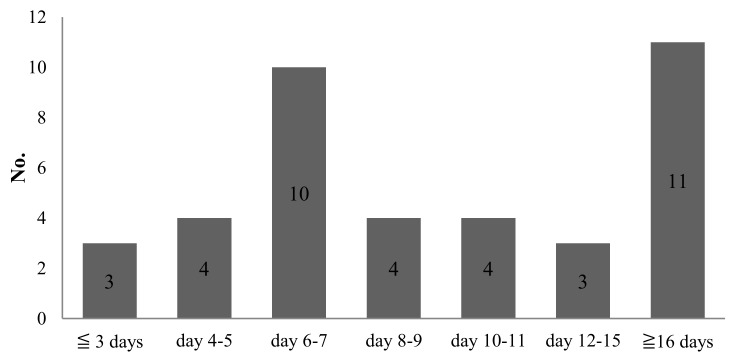
Time from dengue illness onset to fatality.

**Figure 2:**
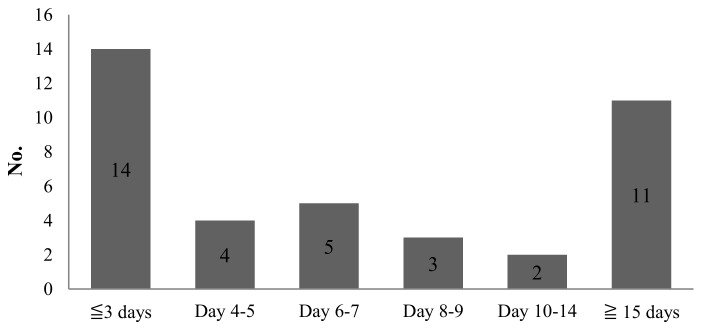
Time from hospital presentation to fatality.

Of the 39 fatal patients (median age: 66 years), the median time from the illness onset and hospital presentation to fatality was 9 and 6 days, respectively. Among these patients, 17 (median age: 69 years) died ≤7 days after the onset of dengue, and 14 (median age: 66 years) died ≤3 days after hospital presentation (Figure 1 and Figure 2).

The occurrence of warning signs ≤72 h after presentation were as follows: abdominal pain, 30.7%; vomiting, 20.5%; drowsiness, 51.2%; gastrointestinal bleeding, 69.2%; pleural effusion, 28.2%; ascites, 15.3%; and concurrent increase in hematocrit level and rapid decrease in platelet count, 16.6%. Leukocytosis was observed in 14 (35.9%) fatal patients on the day of presentation. Of these 14 patients with leukocytosis at presentation, 1 had bacteremia and another experienced candidemia. Thirty-two (82%) fatal patients subsequently developed leukocytosis during their hospitalization. Regarding the complications in the 39 fatal patients, who may have had more than one complication during the entire clinical course, hemoconcentration was observed in 30 (76.9%) patients, acute kidney injury in 27 (69.2%), bacteremia in 11 (28.2%), myocardial injury in 11 (28.2%), pneumonia in 7 (17.9%), rhabdomyolysis in 4 (10.2%), and candidemia in 2 (5.1%). Severe hepatitis was found in 6 (21.4%) of 28 patients with AST or ALT. Of the 11 patients with bacteremia, monobacteremia was found in 10 patients and polybacteremia was identified in 1 patient. The median time from presentation to bacteremia was 1 (range: 1–13) day. Eight (72.7%) of the 11 bacteremic patients presented with bacteremia <3 days after presentation. Regarding monobacteremia, *Klebsiella pneumoniae* was isolated from four patients, *Corynebacterium* species from two patients, and *Streptococcus anginosus*, *Proteus mirabilis*, *Bacteroides fragilis*, *Escherichia coli*, and *Acinetobacter baumannii* from one patient each. Candidemia was observed in two patients on hospital days 3 and 20, respectively. Three patients (one with polybacteremia and two with candidemia) received inappropriate empirical antibiotic treatment before the availability of the blood culture results. All these patients received adequate antibiotic therapy within 48 h of the time the culture results were available. Of the 11 patients with dengue who presented with myocardial injury, the median troponin-I level was 2.49 μg/L, ranging from 0.55 μg/L to 42.8 μg/L (reference value: <0.5 μg/L). The characteristics of the 39 fatal patients are shown in Table 2.

### 3.3. Comparison between Survivors (N = 1047) and Non-Survivors (N = 39) (Table 2 and Table 3)

Fatal patients were significantly older and mostly men, and a higher prevalence of diabetes along with hypertension and other comorbidities as well as end-stage renal disease was observed in such patients than survivors. In addition, cough, fever, rashes, and headache were significantly less reported in fatal patients. Regarding warning signs (≤72 h after presentation), fatal patients had significantly higher frequencies of drowsiness, gastrointestinal bleeding, and pleural effusion. A significantly higher incidence of leukocytosis and lower hemoglobin level and platelet count based on the laboratory data at presentation was observed in fatal patients than survivors. Moreover, a significantly higher incidence of leukocytosis, elevated AST and ALT levels, hemoconcentration, acute kidney injury, myocardial injury, bacteremia, and candidemia in addition to a lower platelet count during the course of hospitalization was observed in fatal patients. The multivariate analysis revealed that gastrointestinal bleeding ≤72 h after presentation (adjusted odds ratio (aOR): 20.278; 95% confidence interval (CI): 5.089–84.426), platelet count < 50 × 10^9^ cells/L at presentation (aOR: 5.422; 95% CI: 1.398–21.025), and the presence of leukocytosis (aOR: 12.763; 95% CI: 3.788–43.003) and hemoconcentration (aOR: 55.674; 95% CI: 13.110–236.422) during hospitalization were independently correlated to overall mortality from dengue.

### 3.4. Comparison between Survivors (N = 1047) and Individuals Who Died ≤7 Days after Illness Onset (N = 17) (Table 2 and Table 3)

Significant differences in the demographic information and clinical manifestations between fatal patients (≤7 days after illness onset) and survivors included old age, diabetes and hypertension with other comorbidities, gastrointestinal bleeding and pleural effusion (≤72 h after presentation), as well as bone pain and rashes at presentation. Several conditions, such as leukocytosis and a low platelet count at presentation, were significantly associated with mortality ≤7 days after illness onset. During hospitalization, leukocytosis, elevated AST and ALT levels, and a low platelet count, as well as hemoconcentration, acute kidney injury, bacteremia, and myocardial injury, were associated with increased mortality ≤7 days after the illness onset. The multivariate analysis showed that gastrointestinal bleeding ≤72 h after presentation (aOR: 8.879; 95% CI: 2.024–38.961), leukocytosis (aOR: 19.925; 95% CI: 4.875–81.443), and hemoconcentration (aOR: 71.667; 95% CI: 16.375–313.661) during hospitalization were independent risk factors of death ≤7 days after illness onset.

### 3.5. Comparison between Survivors (N = 1047) and Individuals Who Died ≤3 Days after Presentation (N = 14) (Table 2 and Table 3)

Compared to the survivors, patients who died ≤3 days after presentation had a significantly older age, gastrointestinal bleeding, and drowsiness (≤72 h after presentation), as well as a low platelet count and elevated AST and ALT levels at presentation. Patients who died ≤3 days after presentation had a higher incidence of leukocytosis, a low platelet count, elevated AST and ALT levels, hemoconcentration, acute kidney injury, bacteremia, and myocardial injury during the entire course of hospitalization. The multivariate analysis showed that gastrointestinal bleeding ≤72 h after presentation (aOR: 15.854: 95% CI: 2.911–86.356), platelet count < 50 × 10^9^ cells/L at presentation (aOR: 18.562; 95% CI: 2.521–136.668), and acute kidney injury (aOR: 318.987; 95% CI: 47.053–2162.495) during hospitalization were independent risk factors of death ≤3 days after presentation. 

### 3.6. Risk Scoring Models (Table 3)

**Figure 3:**
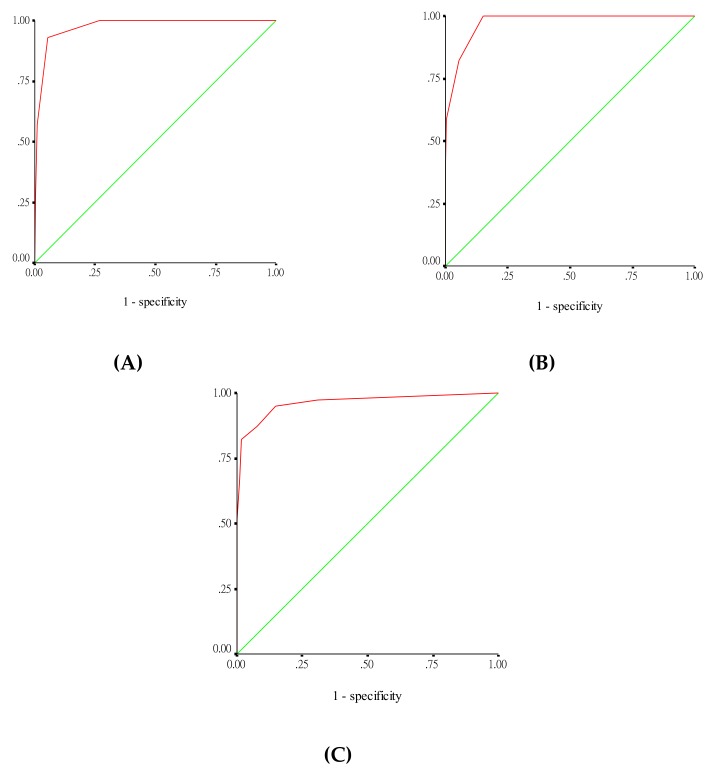
Receiver operating characteristic curve for differentiating survivors from non-survivors: (**A**) survivors versus individuals who died ≤3 days after presentation, (**B**) survivors versus individuals who died ≤7 days after illness onset, (**C**) survivors versus overall fatal patients.

The score for death ≤3 days after presentation model (model-1) included 1 point for gastrointestinal bleeding ≤72 h after presentation and a platelet count of <50 × 10^9^ cells/L at presentation, as well as 2 points for acute kidney injury during hospitalization (scale ranging from 0 to 4). This model was an excellent discriminant between survivors and those who died ≤3 days after presentation (AUC: 0.975), with 2 points as the cutoff level, corresponding to 92.9% sensitivity and 94.7% specificity (Figure 3A). The fatality rates for risk scores of 0, 1, 2, 3, and 4 points were 0%, 0.4%, 9.8%, 50%, and 50%, respectively (*P* < 0.001, Cochran–Armitage trending test).

For deaths ≤7 days after illness onset (model-2), the predictive score included 1 point for gastrointestinal bleeding ≤72 h after presentation and leukocytosis during hospitalization, as well as 2 points for hemoconcentration during hospitalization (scale ranging from 0 to 4). The ROC curve showed an excellent capability to predict fatality ≤7 days after the illness onset, with an AUC of 0.974 (Figure 3B). A cutoff value of 2 points had 82.4% sensitivity and 94.6% specificity for deaths ≤7 days after illness onset. The fatality rates for risk scores of 0, 1, 2, 3, and 4 points were 0%, 2.8%, 7.4%, 37.5%, and 100%, respectively (*P* < 0.001, Cochran–Armitage trending test).

For the overall fatality model (model-3), the predictive score included 1 point for a platelet count of <50 × 10^9^ cells/L at presentation and 2 points for gastrointestinal bleeding ≤72 h after presentation, as well as leukocytosis and hemoconcentration during hospitalization (scale ranging from 0 to 7). This scoring model showed excellent discrimination between survivors and non-survivors, with an AUC of 0.963 (Figure 3C). The optimal cutoff value of the risk score for predicting the overall fatality of dengue was 2 points, and the sensitivity and specificity of this cutoff were approximately 94.9% and 85.2%, respectively. The fatality rates for risk scores of 0, 1, 2, 3, 4, 5, 6, and 7 points were 0.1%, 0.6%, 3.8%, 3%, 46.1%, 35.3%, 100%, and 100%, respectively (*P* < 0.001, Cochran–Armitage trending test).

## 4. Discussion

This study first assessed the factors of death ≤7 days after the onset of dengue and ≤3 days after presentation in adult dengue patients. Our dataset includes clinical signs and symptoms at presentation, laboratory results at presentation and over the course of hospitalization, as well as complications during the entire clinical course. In our study population, the mortality rate of dengue was 3.6%. We observed that 43.5% and 35.8% of non-survivors died ≤7 days after the onset of dengue and ≤3 days after hospital presentation, respectively. Remarkably, our study developed three risk scoring models based on presentation and the time after the onset of dengue. Moreover, we identified the predictors of fatality in adults with dengue that can be considered effective measures for patient care to reduce dengue-related mortality and morbidity.

In a study of 34 deaths from dengue, Huang et al. developed the mortality score for dengue fever (ranging from 0 to 5 points) using older age, hypotension, hemoptysis, diabetes mellitus, and a chronically bedridden state for the prediction of 30-day mortality from dengue [29]. However, the study examined pooled clinical signs/symptoms and laboratory data, which may mask some valuable information in the assessment of dengue mortality risk. Furthermore, the study included both children and adult patients. Of note, there may be variations in the clinical manifestations among children and adults with dengue viral infection, which could bias the results. In contrast, our study included only adult patients with dengue and used the data from the time of hospital presentation to the time of hospital discharge (or fatal outcome), reflecting the actual scenario of the entire clinical course of dengue. Apart from the investigation of the predictors of overall mortality from dengue, our study aimed to explore the predictors of death ≤7 days after illness onset and ≤3 days after presentation, and these predictors could help clinicians conduct timely management of patients who may be at high risk of severe dengue and death. 

A single risk scoring model cannot accurately identify patients who are at risk of developing complications during the different clinical phases of dengue [3]. Herein, we developed three risk scoring models based on the illness duration and presentation to assess patients who are at higher risk of mortality from dengue. We established a risk scoring model (model-1) to identify the predictors of death ≤3 days after presentation in patients with dengue. In model-1, the combination of three parameters, including gastrointestinal bleeding ≤72 h after presentation, a platelet count of <50 × 10^9^ cells/L at presentation, and acute kidney injury after hospitalization, using a risk score cutoff value of 2 points, identified fatal dengue cases with a sensitivity of 92.9%. Patients with a risk score of 3 had a mortality rate of 50% during the first 3 days of presentation. By contrast, patients with dengue viral infection who presented within the first week of dengue illness were found to be at higher risk for unfavorable outcomes using model-2, which includes gastrointestinal bleeding ≤72 h after presentation, as well as hemoconcentration and leukocytosis after hospitalization, with good sensitivity (82.4%) and specificity (94.6%) once a cutoff value of 2 points was applied. Notably, clinicians should exert more efforts on patients with risk scores >2 points, who are at mortality rate >35% within the first 7 days of illness. In contrast, patients with a score of 0–1 have a comparatively low (<2.8%) mortality rate. The independent risk factors in model-2 were also the predictors of overall mortality from dengue (model-3), which includes thrombocytopenia (platelet count < 50 × 10^9^ cells/L) at presentation. Remarkably, the scale score for gastrointestinal bleeding and leukocytosis in model-3 was augmented, and this reflects the importance of these variables on survival during hospitalization. Our analysis showed that using a cutoff value of 2 points in model-3 had an excellent sensitivity of 94.9% (AUC: 0.974) for predicting the overall fatality from dengue. Model-3 could be applied to patients with prolonged hospital stays (>7 days) due to dengue. Notably, the overall mortality rate was approximately 46.1% for patients with a risk score of 4. Finally, all the variables in our three risk scoring models can be easily obtained and used in clinical practice, which will assist clinicians in deciding which patients with dengue need hospitalization and in providing timely and effective management to reduce mortality and morbidity.

The occurrence of gastrointestinal bleeding ≤72 h after presentation increased the odds of death in adult patients with dengue throughout the entire clinical course. This finding is in accordance with those from previous studies, which have emphasized the importance of the early recognition of gastrointestinal bleeding to prevent mortality and morbidity [30,31]. In our study, thrombocytopenia (platelet count < 50 × 10^9^ cells/L) was an independent predictor of mortality ≤3 days of presentation and in the overall study population. Although thrombocytopenia is not directly responsible for the risk of bleeding per se in dengue, a significantly lower platelet count of <50 × 10^9^ cells/L exacerbates the bleeding complication, which can lead to an unfavorable outcome [32]. In addition to thrombocytopenia, our earlier study also revealed that older age, as well as end-stage renal disease and previous stroke with comorbidities, were independent predictors of gastrointestinal bleeding in adult patients with dengue [33]. We believe that deaths caused by dengue-related bleeding might be prevented by adopting an appropriate clinical protocol that includes careful assessment and prompt identification of patients who are at risk of gastrointestinal bleeding. Proton pump acid inhibitor therapy may be of benefit and can be considered early in treatment.

Increased vascular permeability is one of the cardinal features of severe dengue and is one of the major causes of shock in dengue [3,34]. Remarkably, our report showed that around 76.9% of non-survivors developed hemoconcentration during the clinical course. In our series, 69.2% of fatal patients had gastrointestinal bleeding, which can worsen hemoconcentration. These findings revealed that intensive monitoring of vital signs is important for the early identification of patients who may be deteriorating, thereby initiating prompt and aggressive intravenous fluid supplement, which is crucial and considered a lifesaving intervention. 

Our study highlights that leukocytosis is an important laboratory predictor of mortality in adult patients with dengue viral infection, in addition to gastrointestinal bleeding and hemoconcentration. Dengue is characterized by thrombocytopenia and leukopenia [3]. Thein et al. reported that 82.2% of dengue patients had an absolute neutrophil count of <1.5 × 10^9^ cells/L [35]. In contrast, an elevated white blood cell count in the peripheral blood is a rare finding in dengue [3]. The presence of leukocytosis shows that the diagnosis of dengue is unlikely. However, in our series, 35.9% of non-survivors had leukocytosis at the day of presentation. The difference in the diagnosis of leukocytosis at presentation and the management due to delay is a challenge for clinicians. Concurrent bacteremia in dengue has been reported in the literature [36,37]. In our report, bacteremia occurred in 72.7% of 11 non-survivors with bacteremia within 2 days after hospital presentation. However, among these patients, only one had leukocytosis. Leukocytosis may have occurred secondary to a bacterial infection, but it can develop due to a variety of etiologies, including volume depletion due to severe plasma leakage and/or bleeding. These findings are important for frontline medical personnel, and leukocytosis may be observed in patients with dengue due to bacterial infection, intravascular volume depletion, or bleeding; in addition, bacteremia can also occur in patients with dengue in the absence of leukocytosis at presentation. Furthermore, 82% of non-survivors in our series subsequently developed leukocytosis, and this result indicated that the presence of leukocytosis during the clinical course is a poor predictor of dengue. Remarkably, inappropriate empiric antimicrobial therapy was found in three fatal patients. This finding highlights the importance of appropriate antimicrobial therapy on outcomes for severe dengue patients with bacteremia (or fungemia).

Organ dysfunction in bacterial sepsis is a well-known poor prognostic factor [38]. In fact, organ involvement is one of the criteria for severe dengue diagnosis in the 2009 WHO dengue scheme [3]. The pathogenesis of acute kidney injury is multifactorial, which includes direct action by the virus, severe plasma leakage, severe bleeding, rhabdomyolysis, and acute tubular necrosis [39]. The prevalence of acute kidney injury has been observed in approximately 30–100% of fatal dengue cases [30,39,40]. Our report revealed that the development of acute kidney injury ≤3 days of presentation is associated with poor clinical outcome. Notably, >70% of all patients with fatal dengue subsequently had acute kidney injury in our series. 

Previous studies have shown that a subsequent infection by another dengue serotype increases the risk of developing severe dengue [41,42]. Serological testing is used to distinguish between primary and secondary infections. In contrast to primary infection, secondary dengue infection results in high levels of anti-dengue IgG, whereas anti-dengue IgM levels are comparatively low during a secondary infection [43]. Even though the data for primary and secondary dengue infection are lacking in our series, a predictive model using more readily available clinical and laboratory characteristics would provide useful information to clinicians. Actually, most hospitals, especially primary healthcare facilities, do not have the capacity to detect anti-dengue antibodies. Thus, the variables used to predict the outcome should be simple and promptly obtained.

Shock is an important sign associated with poor clinical outcome in dengue [1,3]. Acute respiratory failure may develop in patients experiencing shock. The importance of timely and effective volume replacement to prevent progressive dengue severity and shock should be emphasized. In our study, we did not include such an indicator for predicting mortality, because shock usually is a manifestation of late-stage dengue illness and all death cases eventually experienced shock and respiratory failure preceding death. Identification of more reliable and early signs of likely deterioration before shock would be more useful for individual case management for reducing morbidity.

The present study had several limitations owing to its retrospective design. First, the small number of patients who died ≤7 days after illness onset and ≤3 days after presentation may produce a quite low statistical power for the identification of factors associated with dengue-related mortality. Second, the study population comprised adult patients; therefore, the results cannot be generalized to pediatric patients. Third, a validation group was not included in our series. Thus, additional large-scale prospective studies must be conducted to validate these risk scoring models for better generalization in different populations. Nevertheless, the strength of this study is based on our attempt to include detailed clinical signs and symptoms at presentation and complications during the hospital course, and detailed laboratory results at presentation and during the entire clinical course. The present study improved the awareness of clinicians about the factors associated with death among adult patients with dengue viral infection. Furthermore, this is the only study showing the factors associated with death ≤7 days after the onset of dengue and ≤3 days after presentation, and this result provides additional valuable information for appropriate triage and reducing the hospitalization rate of patients with dengue, thereby focusing resources on those who are at the highest risk. 

## 5. Conclusions

In summary, we developed three stratification risk scoring models based on the duration of illness and presentation which have excellent capability in distinguishing survivors from non-survivors. These models can be useful for frontline clinicians who identify those at risk of disease progression and who immediately initiate appropriate treatment to prevent dengue progression and to reduce mortality from this condition.

## Figures and Tables

**Table 1:** Characteristics of adult patients with dengue.

	Included Cases (*N* = 1086)
**Demographic and clinical features**	
Median age (range), years	52 (18–91)
Age > 65 years	191 (17.5)
Male	513 (47.2)
Comorbid condition	
Diabetes alone	58 (5.3)
Hypertension alone	123 (11.3)
Diabetes and hypertension	92 (8.4)
Diabetes, hypertension, and other comorbidity	12 ^a^ (1.1)
End-stage renal disease alone	3 (0.3)
Median time from illness onset to hospital presentation (range), days	3 (1–10)
Severe dengue (2009 WHO)	82 (7.4)
Fatal	39 (3.6)
**Symptoms and signs at presentation**	
Fever	1028 (94.6)
Myalgia	393 (36.2)
Bone pain	463 (42.6)
Rash	386 (35.5)
Headache	445 (41)
Cough	285 (26.2)
Retro-orbital pain	123 (11.3)
Diarrhea	158 (14.5)
Abdomen pain	236 (21.7)
Vomiting	322 (29.6)
Drowsiness	106 (9.7)
Petechiae	251 (23.1)

Data are “number (%)” unless otherwise indicated. WHO = World Health Organization. ^a^ Among the 12 patients with diabetes, hypertension, and other comorbidity, chronic kidney disease was found in 5 patients, end-stage renal disease in 3, end-stage renal disease with ischemic heart disease in 2, as well as chronic kidney disease with ischemic heart disease in 1 patient, and chronic kidney disease with previous stroke in 1 patient.

**Table 2:** Comparison of clinical and laboratory characteristics between survivors and non-survivors.

Variable	Patients Who Survived (*N* = 1047)	Patients Who Died			*P* ^a^	*P* ^b^	*P* ^c^
		≤3 Days after Presentation (*N* = 14)	≤7 Days after Onset of Dengue Illness (*N* = 17)	Overall (*N* = 39)			
**Demographic and clinical features**							
Median age (range), years	51 (18–91)	66 (41–83)	69 (45–86)	66 (33–86)	<0.001	<0.001	<0.001
Age > 65 years	171 (16.3)	7 (50)	9 (52.9)	20 (51.2)	0.004	0.001	<0.001
Male	488 (46.6)	9 (64.3)	11 (64.7)	25 (64.1)	0.281	0.150	0.034
Comorbid condition							
Diabetes alone	54 (5.5)	1 (7.1)	2 (11.7)	4 (10.2)	>0.99	>0.99	>0.99
Hypertension alone	115 (10.9)	3 (21.4)	4 (23.5)	8 (20.5)	>0.99	>0.99	>0.99
Diabetes and hypertension	83 (7.9)	2 (14.3)	3 (17.6)	9 (23)	>0.99	>0.99	>0.99
Diabetes, hypertension, and other comorbidity	5 ^d^ (0.5)	3 ^e^ (21.4)	5 ^f^ (29.4)	7 ^g^ (17.9)	>0.99	<0.001	<0.001
End-stage renal disease alone	1 (0.1)	0 (0)	0 (0)	2 (5.1)	>0.99	>0.99	0.004
Median time from illness onset to hospital presentation (range), days	3 (1–10)	3 (1–8)	1 (1–4)	3 (1–8)	0.803	0.062	0.035
Median time from hospital presentation to fatality (range), days	-	3 (2–3)	3 (2–7)	6 (2–39)	-	-	-
Median time from illness onset to fatality (range), days	-	6 (3–10)	6 (3–7)	9 (3–47)	-	-	-
Severe dengue (2009 WHO)	43 (4.1)	14 (100)	17 (100)	39 (100)	<0.001	<0.001	<0.001
**Symptoms on the day of presentation**							
Fever	996 (95.1)	12 (85.7)	14 (82.4)	32 (82)	>0.99	0.051	0.003
Myalgia	382 (36.4)	4 (28.6)	3 (17.6)	11 (28.2)	0.781	0.131	0.314
Bone pain	452 (43.1)	5 (35.7)	3 (17.6)	11 (28.2)	0.787	0.046	0.070
Rash	383 (36.5)	2 (14.3)	1 (5.9)	3 (7.7)	0.099	0.009	<0.001
Headache	436 (41.6)	3 (21.4)	3 (17.6)	9 (23)	0.173	0.050	0.021
Cough	268 (25.5)	3 (21.4)	5 (29.4)	17 (43.5)	>0.99	0.780	0.016
Retro-orbital pain	122 (11.6)	1 (7.1)	0	1 (2.5)	>0.99	0.244	0.116
Diarrhea	150 (14.3)	4 (28.6)	4 (23.5)	8 (20.5)	>0.99	0.292	0.255
Petechiae	245 (23.4)	2 (14.3)	1 (5.8)	6 (15.3)	0.541	0.142	0.333
**Warning signs ≤72 hours after presentation**							
Abdomen pain	224 (21.3)	4 (28.6)	6 (35.3)	12 (30.7)	>0.99	0.229	0.168
Vomiting	314 (3)	3 (21.4)	6 (35.3)	8 (20.5)	0.573	0.603	0.283
Drowsiness	86 (8.2)	10 (71.4)	8 (47.1)	20 (51.2)	<0.001	>0.99	<0.001
Mucosal bleed							
Gastrointestinal bleed	79 (7.5)	8 (57.1)	10 (58.8)	27 (69.2)	<0.001	<0.001	<0.001
Hemoptysis	19 (1.8)	0	0	0	>0.99	>0.99	>0.99
Gum bleed	90 (8.6)	0	0	0	0.623	0.388	0.068
Clinical fluid accumulation, no./total no. (%)							
Pleural effusion	65/632 (10)	5/14 (35.7)	5/17 (29.4)	11/39 (28.2)	>0.99	0.028	0.002
Ascites	39/411 (9.5)	2/14 (14.3)	2/17 (11.8)	6/39 (15.3)	>0.99	>0.99	0.260
Increase in hematocrit > 20% concurrent decrease platelet count, no./total no. (%)	37/547(6.8)	4/14 (28.6)	4/14 (28.6)	6/36 (16.6)	>0.99	>0.99	>0.99
**Laboratory data on the day of presentation**							
Leukopenia (WBC < 3 × 10^9^ cells/L)	341 (32.5)	2 (14.3)	0 (0)	3 (7.7)	0.248	0.001	<0.001
Leukocytosis (WBC > 10 × 10^9^ cells/L)	10 (0.9)	4 (28.6)	6 (35.3)	14 (35.9)	>0.99	<0.001	<0.001
Median hemoglobin (range) (g/dL)	13.5 (6.4–18) (*n* = 965)	12 (8.6–18.1) (*n* = 12)	13 (8.3–18.1) (*n* = 13)	12.9 (7.5–33.9) (*n* = 37)	0.216	0.442	0.018
Median hematocrit (range) (%)	39.4 (21.4–57.2)	36.6 (25.5–51.9)	38.1 (24.8–51.9)	38.1 (21.8–75)	0.339	0.422	0.052
Median platelet count (range) (×10^9^ cells/L)	100 (1–413) (*n* = 1025)	12.5 (0.3–258)	36 (3.6–258)	44.1 (0.3–258)	<0.001	0.023	0.001
Platelet count < 100 × 10^9^ cells/L, no./total no. (%)	517/1025 (50.4)	12/14 (85.7)	12/17 (70.5)	27/39 (69.2)	>0.99	>0.99	0.856
Platelet count < 50 × 10^9^ cells/L, no./total no. (%)	247/1025 (24)	10/14 (71.4)	9/17 (52.9)	20/39 (51.2)	<0.001	0.010	<0.001
Median AST (range) (IU/L)	70.5 (11–4299) (*n* = 684)	420.5 (66–2884) (*n* = 6)	100 (30–4040) (*n* = 10)	86.5 (20–4040) (*n*= 28)	0.015	0.117	0.250
Median ALT (range) (IU/L)	48 (5–1555) (*n* = 597)	177.5 (27–2491) (*n* = 8)	71.5 (18–2491) (*n* = 12)	67.5 (13–2491) (*n* = 28)	0.037	0.085	0.301
**Laboratory data during hospitalization**							
Leukopenia (WBC < 3 × 10^9^ cells/L))	472 (45)	0	1 (5.9)	6 (15.4)	<0.001	0.001	<0.001
Leukocytosis (WBC > 10 × 10^9^ cells/L)	62 (5.9)	8 (57.1)	13 (76.5)	32 (82)	<0.001	<0.001	<0.001
Median peak hematocrit (range) (%)	40.4 (23.7–57.2)	38.2 (25.5–51.9)	41 (25.5–51.9)	40.8 (23.1–59)	0.300	0.659	0.584
Median nadir platelet count (range) (×10^9^ cells/L)	58 (0.7–307) (*n* = 1033)	13 (3–52) (*n* =13)	15 (5–135)	15.5 (3–303) (*n* = 38)	<0.001	<0.001	<0.001
Platelet count < 100 × 10^9^ cells/L, no./total no. (%)	751/1033 (72.7)	13/13 (100)	17/17 (100)	37/38 (97.3)	0.025	0.010	<0.001
Platelet count < 50 × 10^9^ cells/L, no./total no. (%)	469/1033 (45.4)	12/13 (92.3)	13/17 (76.4)	32/38 (84.2)	0.001	0.013	<0.001
Median peak AST (range) (U/L)	93 (10.5–3392) (*n* = 398)	6577 (966–19,347) (*n* = 5)	5495 (148–19,347) (*n* = 7)	601 (17–19,347) (*n* = 12)	<0.001	0.001	0.006
Median peak ALT (range) (U/L)	73 (7–1729) (*n* = 407)	1637 (758–61,620) (*n* = 6)	928.5 (89–6162) (*n* = 8)	446 (4–6162) (*n* = 13)	<0.001	0.001	0.018
**Complications during the entire clinical course**							
Hemoconcentration (increase hematocrit >20%)	41 (3.9)	7 (50)	13 (76.5)	30 (76.9)	<0.001	<0.001	<0.001
Acute kidney injury	14 (1.3)	10 (71.4)	12 (70.6)	27 (69.2)	<0.001	<0.001	<0.001
Severe hepatitis (AST or ALT >1000 IUL), no./total no. (%)	8/784 (1)	5/10 (50)	6/12 (50)	6/28 (21.4)	>0.99	>0.99	>0.99
Bacteremia	0 (0)	2 (14.3)	3 (17.6)	11 (28.2)	<0.001	<0.001	<0.001
Candidemia	0 (0)	0 (0)	0	2 (5.1)	-	-	0.036
Pneumonia	9 (0.9)	2 (14.3)	1 (5.9)	7 (17.9)	>0.99	>0.99	>0.99
Rhabdomyolysis	6 (0.6)	2 (14.3)	3 (17.6)	4 (10.2)	>0.99	>0.99	>0.99
Myocardial injury	2 (0.2)	5 (35.7)	8 (47.1)	11 (28.2)	<0.001	<0.001	<0.001

Data are “number (%)” unless otherwise indicated. ALT = alanine aminotransferase; AST = aspartate aminotransferase; no./total no. = number of cases/number of overall cases with data available for evaluation; WBC = white blood cell count; WHO = World Health Organization. ^a^ Comparison between survivor and individual who died ≤3 days after hospital presentation. ^b^ Comparison between survivor and individual who died ≤7 days after dengue illness onset. ^c^ Comparison between survivor and total fatal cases. ^d^ Among the five patients with diabetes, hypertension, and other comorbidity, end-stage renal disease was found in 2, as well as chronic kidney disease, chronic kidney disease with ischemic heart disease, and chronic kidney disease with previous stroke each in one. ^e^ Among the 3 patients with diabetes, hypertension and other comorbidity, chronic kidney disease was found in two patients and end-stage renal disease in one. ^f^ Among the five patients with diabetes, hypertension, and other comorbidity, chronic kidney disease and end-stage renal disease with ischemic heart disease each was found in two patients, and end-stage renal disease in one. ^g^ Among the seven patients with diabetes, hypertension, and other comorbidity, chronic kidney disease was found in four patients, end-stage renal disease with ischemic heart disease in two, and end-stage renal disease in one.

**Table 3:** Multivariate logistic regression model for survivors versus non-survivors.

Variable	Adjusted Odds Ratio	95% Confidence Interval	*P*	Coefficient	Risk Score Weight
**Survivors (N = 1047) versus individuals who died ≤3 days after presentation (*N* = 14)**					
Gastrointestinal bleeding ≤72 h after presentation	15.854	2.911–86.356	0.001	2.763	1
Platelet count < 50 × 10^9^ cells/L at presentation	18.562	2.521–136.668	0.004	2.921	1
Acute kidney injury during hospitalization	318.987	47.053–2162.495	<0.001	5.765	2
**Survivor (*N* = 1047) versus individuals who died ≤7 days after illness onset (*N* = 17)**					
Gastrointestinal bleeding ≤72 h after presentation	8.879	2.024–38.961	0.004	2.184	1
Leukocytosis during hospitalization	19.925	4.875–81.443	<0.001	2.992	1
Hemoconcentration during hospitalization	71.667	16.375-313.661	<0.001	4.272	2
**Survivor (*N* = 1047) versus non-survivors (*N* = 39)**					
Gastrointestinal bleeding ≤72 h after presentation	20.728	5.089–84.426	<0.001	3.031	2
Platelet count < 50 × 10^9^ cells/L at presentation	5.422	1.398–21.025	0.015	1.690	1
Leukocytosis during hospitalization	12.763	3.788–43.003	<0.001	2.547	2
Hemoconcentration during hospitalization	55.674	13.110–236.422	<0.001	4.020	2
